# Updating descriptive sensory evaluation of chicken: proposing new protocols and statistical analysis

**DOI:** 10.1016/j.psj.2025.105807

**Published:** 2025-09-08

**Authors:** Claire Siebenmorgen, Marlene Schou Grønbeck, Alexandra Schubert, Jan Gertheiss, Johanna Mörlein

**Affiliations:** a Department of Animal Sciences, Meat Science Group, Faculty of Agricultural Sciences, University of Göttingen, D-37077 Goettingen, Germany; b Danish Technological Institute, Taastrup, Denmark; c Department of Mathematics and Statistics, School of Business, Economics and Social Sciences, Helmut-Schmidt-University, D-22043 Hamburg; d Laboratory for Sensory Analysis and Consumer Research, Faculty of Agricultural Sciences, Georg-August-Universität, D-37077 Goettingen, Germany

**Keywords:** Descriptive sensory evaluation, Biodiversity loss, Cluster analysis, Multiple factor analysis

## Abstract

Descriptive sensory chicken evaluations are mostly conducted using prepared sous-vide breasts. Sustainable poultry systems in response to climate change and biodiversity loss require a closer examination of existing sensory evaluation methods. To address the new requirements, we present sensory evaluation results from two different projects. Using skin, breast, thighs, and minced variations, we demonstrate (1) how chicken carcasses can be evaluated holistically using more than the breast, (2) how animal-specific differences can be eliminated and (3) whether a classical quantitative descriptive analysis (QDA) and the more cost efficient and rapid method of Napping creates better results. This enables us to provide statistical guidance for selecting the sensory evaluation method and design for future sensory evaluations. This opens new evaluation criteria for local breeds and alternative husbandry systems. Furthermore, a new approach for analyzing Napping data is proposed. The implications of our results extend to breeders, policymakers, and scholars, providing information about sensory evaluation of chicken meat to effectively update criteria and methods.

## Introduction

Global meat consumption continues to rise, despite more and more people recognizing environmental and health consequences as well as animal welfare concerns (Springmann et al., 2021). In Europe, chicken meat consumption is expected to reach 25.2 kg per capita by 2025 (European Commission, 2024). This effect is mainly driven by (1) the opinion that chicken meat is healthier, (2) more environmentally friendly, and (3) more cost-effective than red meat (Whitton et al., 2021) (4) no religious restrictions. The current rate for global animal-based food production and consumption is exceeding ecological limits, considering the nine planetary boundaries (Rockström et al., 2024; Tian et al., 2024). The industrialization of animal agriculture is an important driver of biodiversity loss, competition in food production, and greenhouse gas emissions (Bidoglio et al., 2024; Kraham, 2017; Ritchie et al., 2022). The FAO therefore calls upon sustainable livestock farming systems that contribute both to climate goals and to eliminating hunger (FAO, 2024).

Currently, nearly 90 % of broilers in the EU are raised in intensive systems, relying on protein- as well as energy-rich feed and high stocking densities (Augère-Granier, 2019). These vertically integrated systems are designed for economic efficiency, high feed conservation rates, and, finally, meat yield maximization. Whole-chain intensification may reduce greenhouse gas emissions and can maintain economic viability, according to recent research (Cheng et al., 2024). These processes primarily focus on optimizing existing structures, without addressing wider systemic challenges such as: overconsumption, food insecurity, the interdependency of health in ecosystems for animals and humans, as well as loss of agrobiodiversity.

As pressure increases to reform food production amid biodiversity loss and climate change, alternatives are gaining more relevance within poultry production. There is growing consensus in Europe that poultry farming should move toward models that integrate biodiversity, animal welfare, and environmental resilience (Augère-Granier, 2019; Bist et al., 2024; Peter John and Mishra, 2024). One approach is the reintroduction and increasing use of local chicken breeds to improve genetic diversity and the future adaptability to environmental changes (Augère-Granier, 2019; Kpomasse et al., 2023).

Despite their potential, local breeds are often judged as disadvantaged in performance-based evaluations, which are generally focused on feed efficiency, growth rate, and carcass yield (González Ariza et al., 2021; Guarino Amato and Castellini, 2022). When considering effects on climate change, loss of agrobiodiversity, and on one health initiative, the potential of local breeds becomes more relevant. The current definition of chicken meat quality focuses on classical criteria, like breast yield and sous-vide sensory evaluation, yet this does not reflect the diversity of the current poultry market (Tura et al., 2024).

The traditional descriptive sensory evaluation of chicken was developed in the 1940s and standardized in the 1980s, and since then has been focusing primarily on sous-vide prepared breast (Crocker, 1948; Schaffheitle and Light, 1989). New production systems for chicken meat, as a response to climate change and biodiversity loss, should also impact sensory evaluation. We utilized the results of two research projects that investigated sustainable chicken production from different perspectives: local breeds and alternative husbandry systems. The projects were conducted from the (1) Danish Technological Institute – DTI (mEATquality") and the (2) University of Göttingen (“ÖkoGen”). The sensory descriptive analyses included panels of trained humans under ethical clearance. To address the difficulties in comparing results from different projects, we look at the methodological differences and systematically analyze the sensory output. Therefore, we included a whole-carcass evaluation, which examined besides breast, the preparation of skin, thighs, and minced parts, and compared two descriptive sensory methods.

The two main objectives of this paper are:

I. To present updated and holistic sensory evaluation protocols for different parts of the chicken carcass. To the classical and traditional breast evaluation, thighs, minced variations, and skin are added.
II. To compare and discuss two descriptive sensory methods: quantitative descriptive analysis and Napping. A new approach for analyzing Napping data is proposed that can be employed as an alternative to the commonly used Multiple Factor Analysis (MFA).

## Materials and methods

### Danish Technological Institute: project

The Study I_QDAb&s_ conducted by the DTI was done within the framework of the “mEATquality” project, which compared intensive and extensive farms, focusing on genetics, diet, and enrichment. The project investigates three different concepts:

(1) Concept 1: higher welfare-non-organic (Diet x Space Allowance) from the Netherlands, where the first treatment High Roughage (HR) had nearly double the space (39kg/m^2^) compared to standard chickens (21kg/m^2^) and the diet included alfalfa, high in fiber, to support a healthy digestive system. The treatment Non-Roughage (NR) had a higher space allowance (39kg/m^2^), but alfalfa was not added to the diet. The treatment Low Roughage (LR) had a standard space allowance (21kg/m^2^), and the diet included alfalfa.
(2) Concept 2: higher welfare-non-organic (Genetics x Enrichment) from the Netherlands, where one treatment was the breed Label Rouge-Naked Neck S757N (Label E), which had access to enrichment like straw bales and dust-bathing areas. The other treatment was the breed JA787 (JA787) with no enrichment added to their living condition.
(3) Concept 3: from Germany, higher welfare – organic (Genetics x Enrichment) using different breeds, with no enrichment added to their living condition, a Slow growing breed (SG), a Dual-purpose breed (DP), and a Male Layer (ML).

A key aspect of this project was to evaluate the human sensory evaluation of different production systems using a trained panel. The project is funded by the European Union’s Horizon 2020 Research and Innovation Programme under Grant Agreement No 101000344 and runs from September 2022 to March 2026.

The total expenses for the sensory panel were €7,478, and panelists received €20.77 per hour.

Poultry from Concept 1 and 2 were slaughtered at V.O.F. Kapteijns en van Gerven (Diessen, The Netherlands) approx. 35 min away from the production facility. After slaughter, poultry carcasses were chilled for 24 hours at 4°C post mortem, vacuum-packed, frozen, and transported to the DTI (approximately 800 km distance). Poultry from Concept 3 were slaughtered at the Bio Frischgeflügel Roth GmbH & Co. KG slaughterhouse (Witzenhausen, Germany), where they were chilled for 24 hours at 4°C post mortem, vacuum-packed, frozen, and transported to the DTI (approximately 600 km distance). The average slaughter age and weights are listed in Table 1. After arriving at the DTI, the carcasses were stored at −18°C for three to five months before the sensory evaluation.

**Table 1:** Danish technological institute: average carcass weight and standard deviation after slaughtering.

Slaughterweight (g)	HR-WP4	NR-WP4	LR-WP4	Label- E	JA787-NoE	SGxNoE	DPxNoE	MLxNoE
Mean±SD	1700 ±150	1660 ±170	1690 ±140	1390 ±100	1550 ±150	1840 ±430	1680 ±170	760 ±95

HR-WP4, High spacexRoughage; NR-WP4, High_spacexNo_Roughage; LR-WP4, Low_spacexRoughage; Label-E, S7575NxEnchriment; JA787-NoE, JA787-No_Enrichment; SGxNoE, Slow_GrowingxNo_Enrichment; DPxNoE, Dual_PurposexNo_Enchriment; MLxNoE, Male_LayerxNo_Enchriment; SD: standard deviation.

All sensory screenings, training, and data collection were carried out at the sensory laboratory of the DTI. The laboratory is equipped with 18 individual booths, maintained at a constant temperature of 22°C, and lighting conditions following ISO 8589 (ISO, 2010). All booths are equipped with fluorescent daylight lamps, and the laboratory is equipped with ventilation, providing an air exchange rate of six renewals per hour. Each booth is outfitted with a portable iPad 8 (Apple Inc., Cupertino, CA, USA) running RedJade® software (Version 5.1.1, Pleasant Hill, CA, USA).

### Danish Technological Institute: sensory analyses

Before to the sensory evaluation, carcasses were thawed at 3-4°C for 24 hours in a refrigerator (Gram; Model: M660 CXG). The chickens were prepared in an Electrolux Air-O-Stream oven (AOS101EA, Stockholm, Sweden) at a set temperature of 175°C. Before cooking, the wings were removed, and the carcass was weighed. The amount of salt and cooking time were calculated based on the carcass weight (Table 2). The salt was gently rubbed into the breast skin. After cooking, the core temperature was measured (average 70°C). The skin was gently removed by cutting along the sides of the breasts, then cutting the skin in half, removing the edges, and dividing each piece into four pieces. Afterwards, the breast filets and skin were removed intact, and each filet and skin were split into four pieces, each measuring approximately 1.5 × 5 cm. Each panelist received a warm plate coded with three digital numbers and served simultaneously a piece of skin and breast.

**Table 2:** Danish technological institute: cooking time and amount of salt based on carcass weight.

Carcassweight (g)	Cooking time (min.)	Amount of salt (g)
1800	50	3
1600	45	3
1400	40	2.5
1200	35	2.5
1000	30	2
800	25	2
600	20	1

#### Training and panelists

The accreditive (DANAK - 05-0392) sensory panel at DTI was used for this study. The panelists are specialized in evaluating meat, and there was no initial panel screening phase. Eight panelists participated in two three-hour training sessions, resulting in 6 hours of training. The first step was to develop a vocabulary of 20 attributes describing skin and breast (Table 3). The second step was to familiarize the panel with intensities for the different sensory properties of chicken meat, and to assimilate the scoring scale to be used. The study was conducted over a period of three weeks (starting week 44, 2023). At the beginning of each new week, the panel had one hour of additional training to refresh their memory and align as a panel.

**Table 3:** Danish technological institute: attributes and definition for the QDA- analysis of breast and skin.

Class	Attribute	Carving	Definition
Appearance	Color	Breast	The color of the meat
	Bleeding	Breast	Red spots in the meat
Odor	Boiled chicken	Breast	Odor boiled chicken meat
	Sweet	Breast	Sweet odor
Flavor	Boiled chicken	Breast	Flavor of boiled chicken meat
	Sour	Breast	Sour intensity on the tongue
	Sweet	Breast	Sweet intensity on the tongue
	Metal	Breast	Flavor of blood and iron
Texture	Firmness at first bite	Breast	How much force is required to chew the meat with molars at the first bite
	Juiciness	Breast	Juiciness after 5 chews
	Tenderness	Breast	Ease with which the meat is broken down during chewing
	Stringy	Breast	Meat separates into fibres that stick between the teeth
	Crumbly	Breast	Meat breaks into small fine pieces that stick in the molars
Aftertaste	Bitter	Breast	Bitter taste after the sample had been spit out
Appearance	Color	Skin	Color of the skin
Texture	Crispiness	Skin	How crispy the skin is when chewed
	Greasy mouthfeel	Skin	Greasy coating in the mouth
Flavor	Fried chicken	Skin	Flavor of fried chicken meat
	Salt	Skin	Salty flavor on the tongue
	Fat	Skin	Flavor of animal fat
Aftertaste	Umami	Skin	Umami taste on the tongue

A QDA in accordance with ASTM-NML 13, ISO 4121, DIN 10964, and DIN 10952 was done. Each session, the panellists evaluated 14 samples randomized by treatment and had a 15-minute break after every five samples. The samples were served eight minutes apart, with panelists seated individually and instructed not to communicate during evaluation. The treatments were evaluated in randomized order, but in the same serving order for the panel, under controlled conditions to prevented interaction between panelists and samples. The panel used a 15 cm unstructured line scale to evaluate the samples. Tap water, sparkling water, unsalted crisp bread (Wasa), and peeled cucumber were used for neutralization between the samples. In total, the trained panel completed 8 hours of training (2 × 3-hour sessions, 2 × 1-hour refreshment sessions), followed by 37 hours of data collection.

### University of Göttingen: project

The sensory analyses conducted by the University of Göttingen on local chicken crossbreeds were carried out within the framework of the 'ÖkoGen' project. The project is funded by the Federal Ministry of Agriculture, Food and Home Affairs (BMLEH) based on a resolution of the German Bundestag. The project is managed by the Federal Office for Agriculture and Food (BLE) within the framework of the Federal Organic Farming Program. The project runs from November 2022 to September 2025 in compliance with applicable national and international regulations, specifically adhering to EU Directive 63/2010. Approval for the animal study was granted by the local authorities in Lower Saxony (file number 33.19-42502-04-00-00204). All procedures involving animals were performed following the principles of Good Veterinary Practice. The total expenses for all sensory panel studies amounted to €5,254, with panelists receiving a compensation of €17 per hour.

The studies investigated three crossbreeds bred from three local German breeds, Altsteirer (ALT), Bielefelder Kennhuhn (BIE), and Ramelsloher (RAM), and the commercial hybrid strain White Rock (WR) (Lohmann Breeders GmbH, Cuxhaven, Germany), resulting in the following:

(1) Altsteirer x White Rock (ALTxWR)
(2) Bielefelder x White Rock (BIExWR),
(3) Ramelsloher x White Rock (RAMxWR).

Each group of crossbreeds was reared in mobile housing systems (4 m² outdoor space per bird) at Bio Frischgeflügel Roth GmbH & Co. KG. Following a starter diet, the crossbreeds were divided into two feeding subgroups from the 9th week of age. The first subgroup was gradually given a diet supplemented with alfalfa: starting with 10 % from week 9, increasing to 15 % in week 12, and then 20 % from week 13 onward (this group is referred to as **_**ALF). The second subgroup continued receiving the same feed mixture as in week 8 (wheat, millet, and oats) and is referred to as _NOR (normal feeding). Due to an incident, nearly all animals in the ALTxWR_NOR subgroup died; therefore, only the ALTxWR_ALF subgroup could be studied further. In addition to the crossbreeds, another group of chickens (served as a zero-control group) was reared in the same mobile housing systems, getting the same feed as the second crossbreed subgroup:

(4) Hubbard JA57 x Coloryield (STD).

All animals were slaughtered at the Bio Frischgeflügel Roth GmbH & Co. KG slaughterhouse (Witzenhausen, Germany), weighed (Table 4), chilled for 24 hours at 4°C post-mortem, vacuum-packed, labeled, and transported to the University of Göttingen (approximately 30 km distance). There they were stored frozen at −20°C for 2 months.

**Table 4:** University of Göttingen: average carcass weight ± standard deviation after slaughtering.

Slaughterweight (g)	ALTxWR_ALF	BIExWR_NOR	BIExWR_ALF	RAMxWR_NOR	RAMxWR_ALF	STD_NOR
Mean±SD	810±74	918±140	837±86	851±98	836±190	1648±185

ALTxWR, Altsteirer x White Rock; BIExWR, Bielefelder x White Rock; RAMxWR, Ramelsloher x White Rock; STD, Standard Hybrid Hubbard JA57 x Coloryield; _NOR, fed with normal feed; _ALF, fed with alfalfa; SD: standard deviation.

All sensory screenings, training, and data collection were carried out at the sensory laboratory of the Faculty of Agricultural Sciences at the University of Göttingen. The laboratory is equipped with 10 individual booths, maintained at a constant temperature of 21°C, and features adjustable lighting conditions following ISO 8589 (ISO, 2010). All booths are equipped with fluorescent daylight lamps, and the laboratory is equipped with ventilation, providing an air exchange rate of six renewals per hour. Each booth has an iPad 9 (Apple Inc., Cupertino, CA, USA) for data collection with EyeQuestion® software (Version 5.2; Elst, Netherlands). If necessary, warming plates (WPS 857, Rommelsbacher, Dinkelsbühl, Germany) are used with each booth to ensure consistent sample temperature during evaluation.

### University of Göttingen: sensory analyses

In total, six sensory studies were conducted (Table 5).

**Table 5:** University of Göttingen: overview of the six descriptive analyses using four breeds: (1) BIExWR_NOR, (2) BIExWR_ALF and (3) RAMxWR_NOR, (4) RAMxWR_ALF additional breeds are given in the table.

Acronym	Method and material	Additional breeds	Number of panelists
Study II**_m_**_QDA_**_b_**	QDA **m**inced **b**reast.	STD	12
Study II**_m_**_QDA_**_t_**	QDA **m**inced **t**highs	STD	12
Study III**_m_**_Napping_**_t_**	Napping **m**inced **t**highs	STD	12
Study III**_m_**_Napping_**_b_**	Napping **m**inced **b**reast	STD	12
Study IV**_s_**_QDA_**_b_**	QDA **s**ous-vide **b**reast	ALTxWR_ALF	10
Study IV**_s_**_Napping_**_b_**	Napping **s**ous-vide **b**reast	STD ALTxWR_ALF	12

ALTxWR, Altsteirer x White Rock; BIExWR, Bielefelder x White Rock; RAMxWR, Ramelsloher x White Rock; STD, Standard Hybrid Hubbard JA57 x Coloryield; _NOR, fed with normal feed; _ALF, fed with alfalfa.

#### Panel recruitment and training

To select panelists, 15 individuals were invited to the sensory laboratory. They participated in a panel screening, consisting of three tests: odor discrimination tests, odor identification tests, and identification tests for the basic tastes. Twelve panelists were selected and underwent training over four weeks, resulting in a total of 12 hours of training. Panel performance was monitored during each session to account for individual differences and optimize training approaches. Odor identification was trained using a protocol adapted from Mörlein et al. (2014) with seven substances: Vanillin (sweet, 0.05 %), Butyric acid (rancid butter, vomit-like, cheesy, sweaty, 0.75 %), Diacetyl (buttery, fresh butter, 1.50 %), Lemon oil (lemony, 1.50 %), Cooked potato (0.75 %), S-carvone (caraway-like, 1.0 %), and Eugenol (clove-like, 1.0 %). These solutions were prepared as follows: For a 1.5 % solution, 15 µL of the liquid substance was mixed with 985 µL of propanediol and vortexed for 30 seconds. Then, 20 µL of the solution was pipetted onto an olfactory strip. For 1.0 % and 0.75 % concentrations, 10 µL and 0.75 µL of the substance, respectively, were used with the same procedure. The 0.05 % vanilla strip was prepared by dissolving 25 mL of vanillin in 50 mL of propanediol, followed by applying 20 µL of the solution to the strip. Basic taste identification was trained for the basic tastes Sucrose (sweet), Monosodium glutamate (umami), and Coffein (bitter). The performance is shown in Table 6.

**Table 6:** University of Göttingen: panel performance for odor identification for 7 odors and basic taste thresholds for umami, sweet and bitter.

	Odor identification^1^	Sweet^2^ (g sucrose/L)	Umami^2^ (g monosodium glutamate/L)	Bitter^2^ g coffein/L
Mean	6.9	7.1 (4.32)	6.2 (0.37)	6.6 (0.14)
Minimum	6	5 (1.56)	5 (0.24)	3 (0.07)
Maximum	7	9 (12)	8 (0.70)	9 (0.27)

1 According to Mörlein et al. (2014).

2 According to ISO (2011).

#### Preparation of meatballs

To reduce animal individual differences in Study II_mQDAb_, Study II_mQDAt_, Study III_mNappingt_, and Study III_mNappingb_, meat balls were prepared. Therefore, skin, bones, and tendons were removed, thigh (musculus femoris) and breast meat (musculus pectoralis major) were minced separately using a KitchenAid grinder (KitchenAid, Michigan, USA) equipped with a 3 mm diameter plate, set to speed level 4 for 20 seconds. No binding agents or seasonings were added. For each category (breed and feeding), a minced meat mixture was produced from three carcasses. They were manually formed with approximately 8 g each and placed into 50 mL Duran® beakers. The meatballs were steamed in an MKN Hans Dampf combi-steamer (MKN GmbH & Co. KG, Wolfenbüttel, Germany) at 100°C for 10 minutes until the core temperature reached 70°C, ensuring the elimination of potential Salmonella. Each sample was labeled with a three-digit randomized code.

#### QDA minced: Studies II_mQDAb_ and II_mQDAt_

During the training phase, panelists evaluated meatballs made from pork, beef, and chicken to identify attributes specifically characteristic of poultry. For the training, meatballs from STD samples were used as a reference for attribute development, and reference intensities were anchored for each attribute. Through progressive training, the panel reduced the number of attributes to eight odor and eight taste references. Texture evaluation was intentionally excluded because of the mincing process. The finalized list of attributes and their references is presented in Table 7. Solid reference materials were presented to panelists at each training session in 50 mL Duran® beakers, while liquid reference solutions were served in 25 mL Melipul disposable medication cups (SCHWARZ, Isny, Germany). In total, the panelists underwent 6 training sessions (2 hours each) followed by 2 data collection sessions (2 hours each).

**Table 7:** University of Göttingen: attributes and references for Studies II_mQDAb_ and II_mQDAt_ for the QDA- analyses of minced meat balls.

Class	Attributes	References and preparation		Presented amount	
Odor	Sour		Vinegar (JEDEN TAG: Tafelessig aus Branntwein)		30 ml
	Fatty		Fat from cooked chicken meat (stewed in tap water at 89-95°C for 1 h)		15 ml
	Meaty		Cooked chicken breast (vacuum sous-vide stewed in tap water at 70°C for 1 h)		10 g
	Sweet		Fresh vanilla (OSTMANN, Feine Vanille)		2 g
	Cabbage		Cabbage soup 1 h cooked cabbage (500 g cabbage, 1.5 L tab water)		40 mL
	Pepper		White pepper (LEBENSBAUM: Pfeffer weiß ganz)		15 g
	Umami		Black Soysauce (SEMPIO, Soy Sauce Black,)		30 mL
	Kokumi		Black bean paste (ASSI: korean black bean paste Jjajang)		10 g
Taste	Meaty		Cooked chicken breast (vacuum sous-vide stewed breast meat in tap water at 70°C for 1 h)		10 g
	Sour		Citric acid solution (0.2 g/L in tap water) DIN 8586		40 mL
	Cabbage like		Cabbage soup 1 h cooked cabbage (500 g cabbage, 1.5 L tab water)		40 mL
	Fatty		Fat from cooked chicken meat (stewed in tap water at 89-95°C for 1 h)		15 ml
	Umami		Mononatriumglutamat (0.3 g/L in tap water) DIN 8586		40 ml
	Sweet		Saccarose (6 g/L in tap water) DIN 8586		40 ml
	Pepper		White pepper (LEBENSBAUM: Pfeffer weiß ganz)		15 g
	Bitter		Coffein (0.2 g/L in tap water) DIN 8586		40 ml

The data for Study II_mQDAb_ was collected on November 12, 2024, and the data for Study II_mQDAt_ was collected separately on November 14, 2024. Attribute intensities were rated using a 15 cm line scale (Gomide et al., 2021). The scale ranged from 0 cm (low intensity, anchor "0″) to 15 cm (high intensity, anchor "100″) for each attribute. At the start of each session, a 5-minute calibration period was provided to familiarize oneself with the references. During each session, samples were presented every 7 minutes and placed on warming plates inside the sensory booths to maintain a consistent meatball temperature (*M* = 62°C). Samples were presented in a randomized order to avoid so-called *first sample effects* and *carry over effects*.

#### Napping minced: Studies III_mNappingb_ and III_mNappingt_

The Napping training consisted of six hours. Panelists were taught the principles of Napping, including the use and interpretation of the Napping-Map and dimensions. Panelists first mapped pork, beef, and chicken meatballs to practice verbalizing sensory differences using the Napping dimensions. As training advanced, only chicken samples were used. In the final training sessions, panelists mapped samples from STD and selected crossbreeds. The final data collection took place in two sessions: Study III_mNappingt_ on November 19, 2024; Study III_mNappingb_ on November 21, 2024. In each session, five samples were evaluated in repetition. To prevent sensory fatigue, samples were randomly divided into two batches of five. Panelists evaluated the first five samples within 20 minutes, followed by a 15-minute break, and then assessed the second batch within another 20 minutes. This approach ensured all samples were maintained at a consistent serving temperature (*M* = 50°C) on warming plates. In what follows, the total of ten samples/products evaluated are denoted by P01-P10, with P01 & P06 corresponding to replicates of STD, P02 & P07 to BIExWR_NOR, P03 & P08 to RAMxWR_NOR, P04 & P09 to BIExWR_ALF, and P05 & P10 to RAMxWR_ALF.

#### Preparation of sous-vide meat

After removal from the freezer, the meat cuts were thawed in a refrigerator at 4°C for 12 hours. Once thawed, the vacuum-sealed bags were removed, and the breast fillets (musculus pectoralis major) were patted dry using sterile disposable cloths. Each fillet was then vacuum-sealed into specialized sous-vide bags (Allpax, Papenburg, Germany) using a chamber vacuum sealer (GGM Gastro International, Ochtrup, Germany) set to level 22. The vacuum-sealed fillets were subsequently cooked in a water bath (Bartscher, Salzkotten, Germany) using a fusion chef sous-vide precision cooker (Julabo, Seelbach, Germany) set to 70°C. Due to differences in individual breast weights and thickness, including inner fillet (ALTxWR_NOR *M* = 52.70 g; ALTxWR_ALF *M* = 49.50 g; RAMxWR_NOR *M* = 53.21 g; RAMxWR_ALF *M* = 50.01 g BIExWR_NOR *M* = 54.72 g; BIExWR_ALF *M* = 52.35 g; STD_NOR *M* = 264.22 g) the breast fillets from all crossbreeds were cooked sous-vide at 70°C for 20 minutes, whereas STD breast fillets were cooked for 1 hour. After the cooking, the fillets were removed from the Sous-vide bags and placed on a cutting board. Each fillet was divided into three equally sized pieces measuring 1.5 × 5 cm. To standardize the distribution of samples among panelists and minimize animal-specific variability, each panelist received corresponding sections (upper, middle, or lower) from the breasts of two different animals within the same breed. The meat samples were then placed in preheated 50 mL Duran® beakers, maintained at 50°C, and labeled with a three-digit randomized code. For the sous-vide study, four training sessions (2 hours each) and 1 data collection session (2 hours) were conducted under controlled conditions.

#### QDA sous-vide: IV_sQDAb_

Attribute intensities were set as in Studies II_mQDAb_ and II_mQDAt_. During the 4-hour training session, the panelists agreed by consensus on a list of 13 attributes related to odor, texture, and taste, each represented by a suitable reference (Table 8). Each QDA session was preceded by a 5-minute calibration phase, allowing panelists to familiarize themselves with the references and the STD sample. Samples were served in a randomized order with repetitions in 50°C pre-heated Duran® beakers and placed on warming plates with an average serving temperature of *M* = 49.19°C. I. In each session, five samples (Table 5) were evaluated in repetition. Panelists evaluated each sample within 6 to 8 minutes and were given a 10-minute break after assessing the first five samples to reset and neutralize their sensory perception. Although efforts were made to minimize temperature loss, slight decreases were unavoidable due to the cutting and handling of the sous-vide-cooked samples before serving.

**Table 8:** University of Göttingen: attributes and references for Study IV_sQDAb_ for the QDA- analyses of sous-vide-cooked breast meat.

Class	Attributes	References and preparation	Presented amount
Odor	Sour	Vinegar (JEDEN TAG: Tafelessig aus Branntwein)	30 ml
	Sweet	Fresh vanilla (OSTMANN, Feine Vanille)	2 g
	Cabbage	Cabbage soup 1 h cooked cabbage (500 g cabbage, 1.5 L tab water)	40 mL
	Stable		-
	Bloody		-
Texture	Strength (Force required to bite through the piece with the incisors)	0 % Processed cheese (JEDEN TAG: Schmelzkäse) 50 % Gouda (JEDEN TAG: Gouda Holland) 100 % Carrot	10 g 5 g 5 g
	Juiciness (Amount of liquid released during the first 6 chews)	0 % Beef jerky (JACK LINKS: Beef jerky original) 100 % Fresh apple	10 g 10 g
	Tenderness (Force used to chew the sample - until it can be swallowed)	0 % Beef jerky (JACK LINKS: Beef jerky original) 100 % Fresh banana	10 g 5 g
Taste	Sweet	Saccharose (6 g/L in tap water) DIN 8586	40 ml
	Fatty	Fat from cooked chicken meat (stewed in tap water at 89-95°C for 1 h)	15 ml
	Metallic	Crushed iron pill (DOPPELHERZ: Eisen + Vitamin C)	5 g
	Umami	Mononatriumglutamat (0.3 g/L in tap water) DIN 8586	40 ml
	Bitter	Coffein (0.2 g/L in tap water) DIN 8586	40 ml

#### Napping sous-vide: Study IV_sNappingb_

The training for the Napping procedure of sous-vide chicken was equal to the minced preparation (Studies III_mNappingb_ and III_mNappingt_), and took four hours. The training sessions were conducted with STD samples to ensure that panelists comprehended the process of mapping samples and defining sensory dimensions. The final data collection was done on December 5, 2024, with 10 samples presented in duplicates to the panelists.

The samples were divided into two groups each consisting of five samples. Although the target serving temperature was 50°C, logistical constraints, particularly the time-consuming task of cutting sous-vide breast portions, resulted in an average serving temperature of *M* = 42.1°C.

## Statistical analysis

### Quantitative descriptive analysis data

The sensory data obtained from the QDA studies were analyzed using linear (mixed) models, analysis of variance (ANOVA)/F-tests, pairwise t-tests, and confidence intervals. Specifically, we used common one- or two-way ANOVA for panel-averaged evaluations to check for significant effects of breed/feeding, followed by pairwise t-tests and graphical evaluations of group-specific means and confidence intervals. When analyzing panelist-level data, we additionally included subject-specific fixed or random effects to account for within-panelist associations. All calculations were done using the statistical software R version 4.5.0 (R Core Team, 2025). Mixed models were fit using the add-on package lme4; see, Bates et al. (2015). For graphics, we used ggplot2; see, e.g., Wickham (2016); ggforce; see, e.g., Pedersen (2024), and lattice; see, e.g., Sarkar (2008) for further details.

### Napping data

The standard approach to analyzing Napping data is the so-called MFA implemented in the R-package FactoMineR and with further helpful functions in SensoMineR; also see, e.g., Lê et al. (2023) and below. As an alternative, we propose an approach based on pairwise distances and cluster analysis. The idea behind the new strategy is as follows. Since the orientation of the coordinate system in which the panelists place the products is arbitrary, the main information to use is the pairwise distances between the ten products considered. Given those distances for a panelist, they are invariant against rotations of the (two-dimensional) coordinate systems. If two products are very far apart, the panelist considers them to be very different; if they lie close together, the panelist evaluates them to be very similar. Once the pairwise distances are calculated for each panelist, we propose averaging them across the panel to obtain a single distance matrix. Those distances can then be input to a hierarchical cluster algorithm of choice to form clusters of similar products. We use agglomerative clustering with average linkage and Euclidean distances as implemented in the R package cluster; see, e.g., Everitt and Hothorn (2011) for further details. The found clusters can then be interpreted using the product descriptions provided by the panelists, e.g., through word clouds as available in the R package wordcloud (see Fellows, 2018), which illustrate the descriptions most often found in the respective cluster. Furthermore, the clusters can be compared with the product features not used for clustering, such as the breed/feeding type. To evaluate the associations between cluster membership and breed/feeding in a statistically sound way, we use Fisher’s exact test, which is especially suited for testing associations in contingency tables with small sample sizes.

The basis of MFA of Napping data is to describe each product by a (*2* m)-dimensional vector, where *m* is the number of panelists. This vector contains all the x- and y-coordinates of the respective product according to the *m* panelists. MFA then determines the main directions of variability and projects the (appropriately weighted) data into a lower-dimensional subspace, typically ℝ^2^, where the positions of the products can be drawn and interpreted. So-called confidence ellipses obtained through bootstrapping (Dehlholm et al., 2012) can be used to illustrate uncertainty about the exact locations. The positions of and hence distances between products in ℝ^2^, however, are only approximations (remember, the results of MFA are a projection in a lower-dimensional subspace). We may instead calculate the distances between the products in the original (*2* m)-dimensional space (in fact, if the number of products *n* is smaller than *2* m, the data vectors span an *n*-dimensional subspace of ℝ^(2m)^). Those distances can also be used for clustering, instead of starting with the distances per panelist as described above. However, the rest of the procedure is analogous to the abovementioned procedure.

## Results and discussion

Statistical analysis of panel-averaged sensory evaluations for the Study I_QDAb&s_ revealed significant group differences for many attributes, for both breast and skin (Table 9).

**Table 9:** Danish Technological Institute: summary of p-values for the attributes from Table 3 when testing for overall breed/feeding group differences (panel-averaged evaluations) for the Study I_QDAb&s_.

Class	Attribute	*p*-value	N	Sig
Breast				
Appearance	Color	0.00086	140	***
	Bleeding	0.00097	140	***
Odor	Boiled chicken	<0.00001	140	***
	Sweet	0.10760	140	
Flavor	Boiled chicken	0.00001	140	***
	Sour	0.00001	140	***
	Sweet	<0.00001	140	***
	Metal	0.36202	140	
Texture	Firmness at first bite	0.00001	140	***
	Juiciness	<0.00001	140	***
	Tenderness	0.00002	140	***
	Stringy	0.00017	140	***
	Crumbling	0.00041	140	***
Aftertaste	Bitter	0.00006	140	***
Skin				
Appearance	Color	<0.00001	140	***
Texture	Crispiness	<0.00001	140	***
	Greasy mouthfeel	<0.00001	140	***
Flavor	Fried chicken	<0.00001	140	***
	Salt	<0.00001	140	***
	Fat	0.00005	140	***
Aftertaste	Umami	<0.00001	140	***

Sig, significance *: *p* < 0.05. *, p < 0.01 **, *p*< 0.001**.

**Fig. 1:**
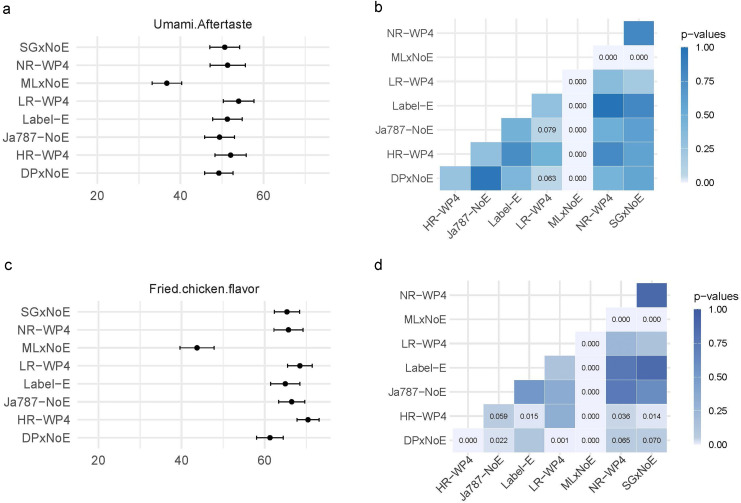
Danish Technological Institute: panel mean evaluations according to QDA of skin (for the Study I_QDAb&s_) for attributes “Umami Aftertaste” (A) and “Fried Chicken Flavor” (C), together with 95 % confidence intervals. B, D give the respective results of pairwise t-testing. HR-WP4, High spacexRoughage; NR-WP4, High_spacexNo_Roughage; LR-WP4, Low_spacexRoughage; Label-E, S7575NxEnchriment; JA787-NoE, JA787-No_Enrichment; SGxNoE, Slow_GrowingxNo_Enrichment; DPxNoE, Dual_PurposexNo_Enchriment; MLxNoE, Male_LayerxNo_Enchriment.

Fig. 1 exemplarily shows the panel's mean evaluations for skin and attributes “Umami Aftertaste” (A, top left) and “Fried Chicken Flavor” (C, bottom left), together with 95 % two-sided confidence intervals.

In addition, the right subplots display the results of pairwise t-tests. For instance, it is observed that the mean evaluations for MLxNoE, are significantly lower than those for all other groups.

**Fig. 2:**
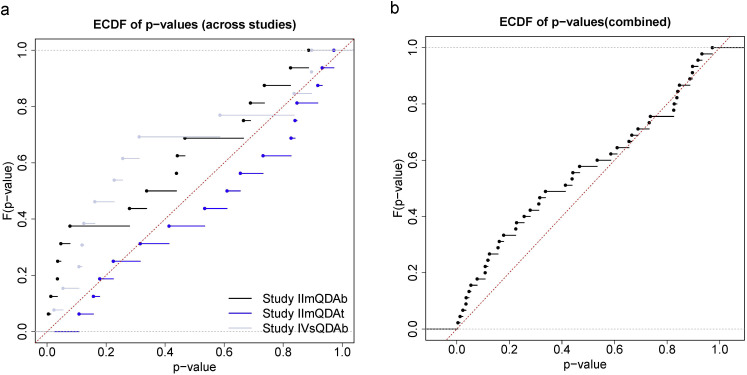
University of Göttingen: empirical cumulative density functions of the *p*-values across the 16 attributes if testing for overall breed/feeding group differences in the linear model with subject-specific fixed effects for Study II_mQDAb_, Study II_mQDAt_, and Study IV_sQDAb_ (A, left), and with p-values combined across studies (B, right).

Given the QDA data from Section 2.4 (Study II_mQDAb,_ Study II_mQDAt_, Study IV_sQDAb_), however, no significant group differences were found for panel-averaged evaluations. Considering the data at the individual level, i.e., the panelist level, a few *p*-values below 0.05, indicating significant breed/feeding group differences, were found for some attributes. However, if we take into account that 16 attributes were evaluated and draw the empirical cumulative distribution function (**cdf**) of the 16 *p*-values resulting from the overall *F*-tests for the 16 attributes (Fig. 2A), this function is relatively close to the diagonal for each of minced breast, minced thigh, and sous-vide. If all the *p*-values are considered together (Fig. 2B), the cdf is even closer to the diagonal. A cdf on the diagonal corresponds to a uniform distribution, which is to be expected for the *p*-values if no (true) differences are given; compare Storey and Tibshirani (2003). As a consequence, the significant group differences found for Studies II_mQDAb,_ II_mQDAt_, and IV_sQDAb_ should be taken with caution.

**Fig. 3:**
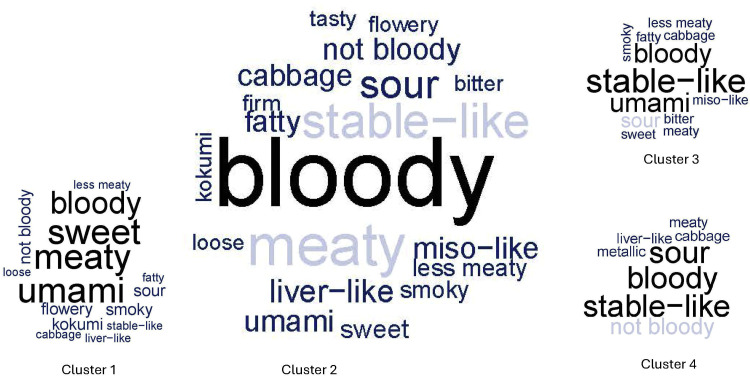
University of Göttingen: wordclouds for the clusters according to the Study III_mNappingt_.

The clusters of the newly proposed clustering approach to analyze Napping data for minced thighs can be interpreted through word clouds of the verbalizations provided by the panelists, as shown in Fig. 3.

**Fig. 4:**
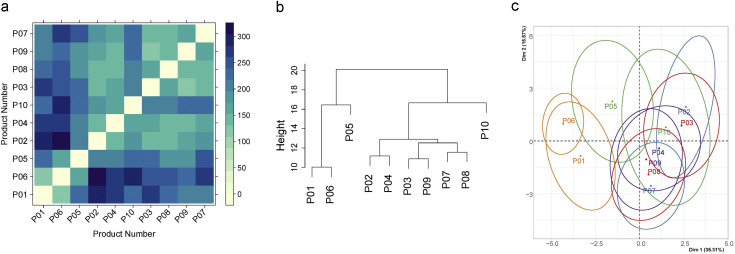
Pairwise, panel-averaged product distances according to Napping of minced thighs/Study III_mNappingt_ (A) and the result of hierarchical, agglomerative clustering (B). (C) gives the product locations in a lower, two-dimensional space according to MFA, together with 95 % confidence ellipses. P04 & P09, Bielefelder x White Rock fed with alfalfa; P02 & P07, Bielefelder x White Rock fed with normal feed; P05 & P10, Ramelsloher x White Rock fed with alfalfa; P03 & P08, Ramelsloher x White Rock fed with normal feed; P01 & P06, Standard Hybrid Hubbard JA57 x Coloryield.

Fig. 4 gives more insights, as subplot 4A gives the pairwise, panel-averaged distances of the ten products; the cluster dendrogram is found in subplot 4B. One large cluster consisting of six products (P02, P03, P04, P07, P08, and P09) and one small cluster consisting of two products (P01 and P06) are identified. Two products (P05 and P10), each forming its own “cluster”, are far from the other products.

Between clusters 1 and 2, the main differences are that cluster 1 products are rather described as sweet and umami, while “stable-like” is more often found in cluster 2. When comparing cluster membership and breed/feeding group, the contingency Table 10 results.

**Table 10:** University of Göttingen: Contingency table of breeds/feeding vs. cluster for the Napping data of minced thighs Study III_mNappingt_.

Breed/feeding group	Product number	Cluster			
		1	2	3	4
BIExWR_ALF	P04, P09	0	2	0	0
BIExWR_NOR	P02, P07	0	2	0	0
RAMxWR_ALF	P05, P10	0	0	1	1
RAMxWR_NOR	P03, P08	0	2	0	0
STD	P01, P06	2	0	0	0

Fisher's Exact Test for Count Data, *p*-value = 0.01587, adj. *p*-value = 0.04761**,** ALTxWR, Altsteirer x White Rock; BIExWR, Bielefelder x White Rock; RAMxWR, Ramelsloher x White Rock; STD, Standard Hybrid Hubbard JA57 x Coloryield; _NOR, fed with normal feed; _ALF, fed with alfalfa.

Both standard products are found in cluster 1, while the largest cluster 2 consists of BIExWR_NOR, BIExWR_ALF, and RAMxWR_NOR. Fisher’s exact test reveals statistically significant dependence between the rows (breeds/feeding) and the columns (clusters), with *p*-value = 0.0159. Considering that the analogous procedure was carried out on minced breast and sous-vide as well (which did not show any significant dependence), the *p*-value for minced thighs should be adjusted accordingly. The most stringent Bonferroni correction yields an adjusted *p*-value of 0.0476, which remains below the 5 % alpha level.

If running the cluster analysis of minced thighs on the distances resulting from the products’ characterizations as 24-dimensional vectors (2 times 12), the resulting clusters are the same as before and, consequently, with the same interpretation. The lower-dimensional representation obtained by MFA is shown in Fig. 3C. Even in the two-dimensional space, it is seen that standard products are relatively far away from the other products and that the six products forming cluster 2 are judged to be somewhat similar by the panel. In Fig. 3C, P10 appears to be similar to the cluster 2 products as well, but this impression is misleading. In the original space, P10 is quite far away (note that the two-dimensional projection in Fig. 3C only accounts for about 50 % of the total variation).

### Sensory evaluation methods

Panels of humans are, by their nature, heterogeneous instruments for generating data. In order to assess whether the relationships observed between products and their human sensory perception are genuine, and not merely the result of uncontrolled variation in responses, the sensory methods must be both sufficient and repeatable. Tura et al. (2024) stated that most sensory evaluations of chicken meat focus on chicken breast, typically prepared using sous-vide or conventional oven methods. The sous-vide cooking allows for minimizing variability in the samples because of precise control of the water temperature and time. However, Park et al. (2020) demonstrated that even small variations among those parameters can greatly affect the physicochemical traits and sensory evaluation of the chicken breast. Grilling or oven baking are known to be complicated to control and to change the sensory attributes through the development of other flavor compounds, e.g., due to the Maillard reaction (Jayasena et al., 2013). Bohrer (2019) showed that sensory attributes of breast and thigh chicken meat only weakly correlate, concluding that sensory evaluation needs to be extended. One solution for an updated sensory assessment of chicken meat is to use minced variation. Mincing allows for the avoidance of animal-individual differences and to evaluate both breast and thigh flavor, excluding texture. Apart from preparation methods, sensory evaluation of chicken faces challenges caused by animal individual factors, such as breed, genotype, sex, age, etc. Such animal individual differences are known and documented for any animal product. Mincing and mixing allow the elimination of such factors.

A QDA enables the sensory scientist to generate a comprehensive sensory description of a product and identify underlying variables. To analyze QDA data, having more than two samples per category is important. For research projects, it is difficult to increase the number of animals, for example, when studying endangered breeds reared under specific conditions. A more cost-efficient and more project-related method for data collection is Napping. Napping is possible for small sample sizes, as is the case in research projects, especially when endangered breeds are analyzed. Using minced meatballs enables the use of Napping for chicken meat evaluation. In contrast to QDA, the criteria used by the panelists are not imposed by the sensory scientist. This is a potential advantage, weighing all the attributes equally. Hand-in-hand with using the new approach to analyze the Napping data, it allows for the investigation of variables of interest in a way that enables sensible conclusions to be drawn, even with limited material.

### Statistical guidance: Napping vs. QDA

The analysis of the studies presented above illustrates that the standard (linear) modeling of QDA data can reveal significant group differences if the sample size is sufficiently large and the effects are substantial; compare Table 9 and Fig. 1. However, it may also happen that (almost) no effects are found that go beyond random fluctuations; compare Fig. 2.

An important point to note is that several attributes are typically considered, and each attribute is tested for group differences. Even if no actual/underlying differences are given for any attribute, a few tests may likely indicate a significant effect, with a *p*-value below the alpha level. That is why those results should be interpreted in a descriptive or exploratory manner rather than in terms of inferential statistics. Alternatively, *p*-values could be adjusted to ensure statistical guarantees. For instance, a procedure that controls the so-called family-wise error rate (FWER) and works relatively generally, but is less conservative than the classical Bonferroni method (where *p*-values are simply multiplied by the number of tests), is the Bonferroni-Holm procedure, which is available in all major statistical software packages. For instance, if applying such a correction to the *p*-values from Table 9, all significant results found there would still be statistically significant (at least) at the 5 % level.

An alternative to QDA is Napping, where raters (panelists or consumers) place the considered products in a two-dimensional coordinate system to indicate similarities and dissimilarities between the products. The standard approach for analyzing Napping data is MFA. For this purpose, each product is described through a high-dimensional vector that collects the product’s coordinates according to each rater (hence having a dimension twice the number of raters). MFA then projects the products into a lower-dimensional space, typically ℝ^2^, where the products’ characteristics can be interpreted in terms of the leading dimensions / main directions of variability. However, a potential problem of this approach is that the proportion of variance reflected in the considered lower-dimensional space can be relatively small. In the study III_mNappingt_ above (Fig. 3C), it was only about 50 %, which means that two products that appear close, i.e., similar in the two-dimensional space, may be quite different in the original higher-dimensional space. This can result in misleading interpretations.

In this paper, we propose an alternative approach to MFA. The starting point is the products’ original distances (i) in the two-dimensional, rater-specific space or (ii) in the higher-dimensional space from above. In the latter case (ii), we can directly calculate pairwise product distances; in the first case (i), we average the rater-specific distances across the panel. Afterwards, a cluster algorithm of choice can be used to form groups of similar products that can be interpreted using the additional verbal/written characterizations provided by the panelists. An essential difference from MFA above is that the products’ original (dis)similarities are used for clustering the products, not some projection in a lower-dimensional space, potentially containing less information. Nevertheless, we also recommend running MFA, as this may give additional information, particularly regarding the main directions of variability found. Furthermore, clusters and MFA results can be compared to validate the findings, i.e., by checking whether the identified groups are also visible in the MFA projections.

Finally, an important question is whether and how Napping can be used for group comparisons, similar to QDA above. The cluster results can be a way to go. As shown above, we can evaluate and statistically test the dependency between cluster membership and breeds/feeding group. In our analysis, we indeed found a significant dependency for minced thighs. This indicates that Napping may provide additional information compared to QDA, also in terms of group comparisons. A crucial point is that we may only (statistically) test dependencies between cluster membership and further product characteristics that were not used for clustering (i.e., calculating the distances between products). Therefore, we must not formally test verbalizations from the panelists versus cluster membership, because at least implicitly, the raters will use those characterizations when positioning the products. If the latter is true, dependency is given by design and nothing to test for. In summary, Napping can be considered a valuable alternative to QDA, even for small sample sizes, that may provide additional information and produce findings where QDA does not.

## Practical implications

Despite the widespread use of sous-vide prepared breasts to evaluate chicken meat, there is general recognition that such sensory evaluations have critical limitations both in their information and their application. This paper gives blueprints for an updated sensory assessment of chicken meat and poultry in general to evaluate the whole carcass:

- To reduce animal-individual factors, minced meat balls can be prepared – separately from breast meat and thigh meat. For one category, at least three animals should be used.
- Using minced meat balls enables the use of Napping as a descriptive sensory analysis.
- Preparing the whole carcass in an oven limits the possibility to randomize the samples between the panelists. At the same time, evaluating a whole carcass can provide essential sensory characteristics for a real-life approach, including the skin as an important part of the chicken for whole carcass marketing.
- In addition to rather descriptive analyses, Napping followed by cluster analysis can also be used to investigate breed/feeding, etc., effects on sensory evaluations in a statistically sound way.

## CRediT authorship contribution statement

**Claire Siebenmorgen:** Writing – review & editing, Writing – original draft, Investigation. **Marlene Schou Grønbeck:** Writing – original draft, Investigation. **Alexandra Schubert:** Writing – review & editing, Writing – original draft, Formal analysis, Data curation. **Jan Gertheiss:** Writing – review & editing, Writing – original draft, Supervision, Formal analysis, Data curation. **Johanna Mörlein:** Writing – review & editing, Writing – original draft, Validation, Supervision, Methodology, Investigation, Conceptualization.

## Disclosures

None of the authors of this paper has a financial or personal relationship with other people or organizations that could inappropriately influence or bias the content of the paper.
